# Considerations in the design of animal infection pilot studies

**DOI:** 10.3389/fcimb.2022.948464

**Published:** 2022-11-03

**Authors:** Thomas R. Laws, Thomas C. Maishman

**Affiliations:** Defence Science and Technology Laboratory (DSTL) Porton Down, Chemical Biological Radiological (CBR) Division, Salisbury, United Kingdom

**Keywords:** sample size, animal study, pilot study, survival, weight change, microbial burden

## Abstract

Ethical research with experimental systems (animals or humans) requires a rationale for the number of subjects to be included in a study. Standard methods for estimating sample size are not fit-for-purpose when the experimenter cannot predict the effect size/outcome with any certainty. These types of studies are often designated “pilot study”; however, there are few guidelines for sample size needed for a pilot study. Here we seek to address this issue. Concerning survival analysis it is noted that the experimenter can adjust the parameters of the experiment to improve the power. We propose that the experimenter needs to consider the “limit of interest” needed to represent an effect that the experimenter would be prepared to defend in terms of scientific or medical interest. Conventional power analysis is then used to estimate the *n* to deliver an alpha (false positive rate) of p < 0.2. This approach provides a balance that can inform a future study, demonstrate a strong effect or dismiss if no effect was observed. Where weight change or infection burden is considered, parametric analysis can be used. Here the main requirement for the pilot study is to establish a meaningful estimate of variability for subsequent power analysis. When considering the confidence intervals for standard deviations, it can be noted that a turning point is reached for *n* of four to six, beyond which we observe diminishing returns, suggesting that sample sizes should be greater than four. Finally, we discuss both the importance in statistical blocking and repeated measures in maximising the usefulness of the pilot study; and the importance of considering and outlining analysis techniques prior to performing the experiment. These findings are intended to be useful in the design of experiments in further prospective research.

## Introduction

The use of animals remains an integral part of infection biology. Knowing how many subjects are required for an experiment is essential for ethical reasons. This is very much reflected in the expectation of funders. Moreover, in the United Kingdom, this is stipulated in law in the conditions of the project licenses under the Animals (Scientific Procedures) Act 1986 (The animals (Scientific procedures) act 1986 (ASPA) and the animals (Scientific procedures) act 1986 amendment regulations 2012 (SI 2012/3039)). In experiments where experimental conditions are compared, there are well-established methods to estimate the numbers of animals required. These ‘power analyses’ can estimate the number of experimental units needed using a known (or aspirational) effect size and known estimates of experimental variability. These are performed with a desired confidence level (*α* which is probability of “false positive” and to the experimenter is the limit of confidence needed to provide adequate conclusions) and statistical power (1–*β*, where *β* is the probability of a “false negative”, or to the experimenter, this is the proportion of random experiments that are fit-for-purpose). However, in prospective science, there is often no sensible estimate for effect size and/or variability. When this is the case often the experimenter will define their experiment as a “pilot study”. We think that it would be appropriate to reflect on what this actually means. A pilot study should be a prospective design that should, at minimum, leave the experimenter in a position to design a further experiment that should allow a hypothesis to be tested at a desired confidence level (usually p <0.05, meaning *p* the likelihood that the data has arisen randomly, providing the conditions of the test are met). A pilot study might be able to provide sufficient evidence to test a hypothesis without further experimentation; however, this is not intrinsic in its design.

Pilot studies have been described as “sometimes involving only a single animal, [these] can be used to test the logistics of a proposed experiment. Slightly larger ones can provide estimates of the means and standard deviations and possibly some indication of likely response, which can be used in a power analysis to determine sample sizes of future experiments. However, if the pilot experiment is very small, these estimates will be inaccurate” (Festing and Altman, 2002). However, there is clearly a need to consider this type of experiment in greater detail. There has also been limited discussion on the sample size required for pilot studies (Browne, 1995; Julious, 2005; Sorzano et al., 2017; Allgoewer and Mayer, 2017), although much of this discussion is confined to clinical trials (Browne, 1995; Julious, 2005). Two studies provide information on sample sizes for animal pilot studies; Soranzo et al. provide some statistical methodology and assumptions, with particular focus on the implications of attempting to detect smaller differences in animal pilot studies (Sorzano et al., 2017). Allgoewer and Meyer provide results from a simulation study, which includes recommendations for categorical and continuous outcomes, and provide a useful comparison to the findings of this study (Allgoewer and Mayer, 2017). It is also important to note that in clinical trials, the scale of effect is frequently influenced by the fact that the comparator group will be standard of care; thus, the novel therapeutic will need to have considerable benefit or a sizeable *N* to be successful. Whilst in animal research, the study designers are able to compare a novel therapy to a true placebo group.

Here this question is considered primarily from the perspective of infection biology. Commonly performed experiments within these laboratories are: endpoint (survival) experiments, experiments where weight change is compared and experiments where infection loads are compared.

## Results and discussion

### Survival

In survival experiments, the aim is to compare the rates or total proportions by which the animals reach a predetermined end point. These end points can vary from the exhibition of clinical signs/weight loss through to characterised levels of pathology that the experimenter is certain cannot be recovered from. These rates are often expressed using Kaplan-Meier (survival) estimates and can be compared through a variety of tests that largely consider the hazard ratio (the ratio of event rates between two groups) (Kaplan and Meier, 1958). Therefore, in order to progress from a pilot study a reasonably precise and accurate estimate of each hazard rate would be needed.

Working from the assumption that the experiment has a partially characterised comparator group, the pilot study can be tailored to maximise the expected difference. The first consideration is whether the intervention (or unknown condition) is likely to increase or decrease the rate of failure. It is harder to visualise improvements upon conditions that already have a low failure rate. Conversely, it is harder to visualise degradations upon conditions that already have a high failure rate. In this way the experimenter should tailor any control group to maximise the expected information gained about the studied effect. In survival analysis, failure events are more informative than survivors and this means different group sizes are needed for different designs. We consider three experiment types:

• Aspiring for greater survival. Should a novel therapy be compared to no treatment, the experimenters should consider engineering the control condition where the failure rate is higher and/or monitor survival for longer. This is the most powerful study because the established group will already provide the greatest number of events.
• Loss of function. Where a loss of function is characterised, the experimenters should consider engineering the control groups to diminish failure rate and/or capture more time-points while the control group decays, thus reducing the granularity.
• Neither expectation. Should two treatment groups be compared with no bias in anticipated outcome, the experimenter should look to maintain a balance on these two approaches.

**Figure 1:**
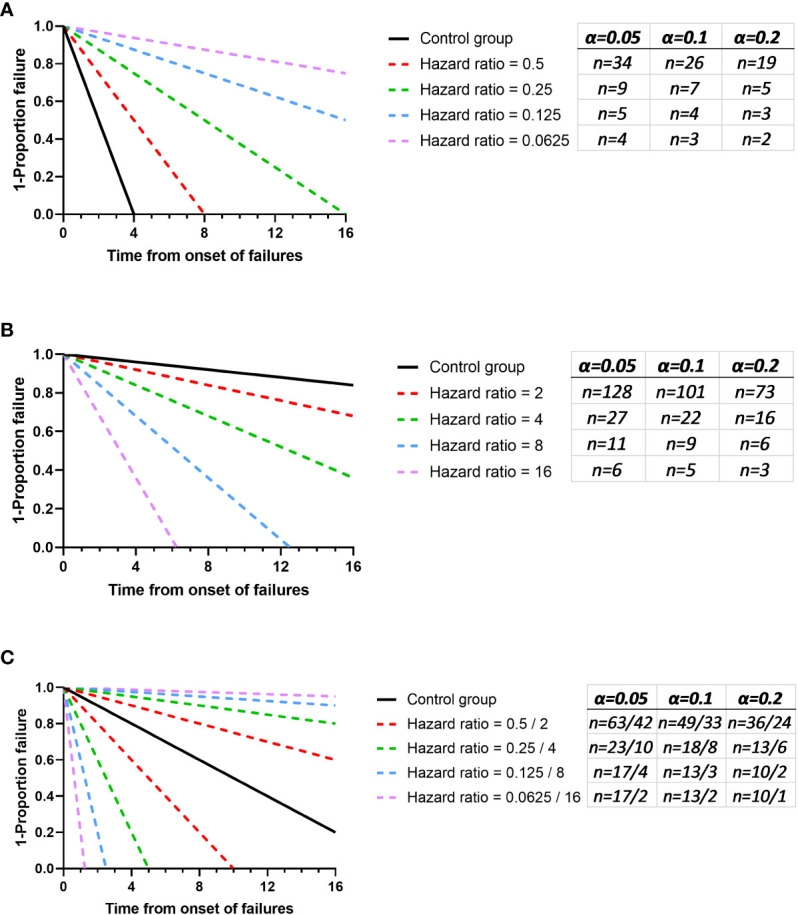
The expected values for n to visualise a significant difference with 80% power and 5%, 10% and 20% alpha. Fixed control groups have been used in each panel and comparator groups with varying effect sizes were used. The sample size calculated for each comparison are added in red. **(A)** shows an experiment where the test group is expected to improve outcome and here the control is considered to have a median survival of 2 time units post onset of failures. **(B)** shows an experiment where the experimental group is likely to increase the failure rate and here the control is considered to have a median survival of 50 time units post onset of failures. **(C)** shows an experiment with no bias in aspiration outcome and here the control is considered to have a median survival of 10 time units post onset of failures. Graphs were generated using Graphpad PRISM V8.0 (San Diego), using the ‘plot line’ function, where the plots of y = a+bx were used and the power analysis was performed using PS: Power and Sample Size Calculation V3.0 (https://biostat.app.vumc.org/wiki/Main/PowerSampleSize). The software uses the method described by Schoenfeld et al. (Schoenfeld and Richter, 1982).

For illustrative purposes, we have investigated these three scenarios using power analysis software to gain an understanding for the types of difference that might be found with varying sample sizes (**Figure 1**). Consistent with expectations, the lowest number of animals are required for experiments where there is an expectation to improve outcome because there are a greater number of failures. Least power was observed where the control group had the least failure in the “loss of function experiment”. These investigations have some utility in managing the experimenter’s expectation with regards to how much of a difference this initial experiment might be able to identify as a “probable real effect”. However, as discussed earlier, this is not the primary function of the pilot study. The pilot study should predict a likely effect size that can then be used to estimate the number of animals required to test the hypothesis at a desired significance level. In this way it is generally wrong for the experimenter to consider the lowest effect size that they may have interest in, a “limit of interest” (i.e. an effect size that would be pursuable for a novel therapeutic, or that would indicate the role of a factor in disease) and perform a conventional sample size calculation for this value. This is reasoned because, without prior knowledge of outcome the effect size may be far in excess of this “limit of interest”. Where this is the case then many participants will have been included unnecessarily. We propose that this limit of interest might be used however with a relaxed α rejection rate. Typically *p* < 0.05 is the threshold used in the biological sciences, denoting a less than 1 in 20 likelihood that the data had arisen randomly. In a pilot study the goal is not to evaluate the hypothesis *per se* but to consider if a course is worth pursuing. We propose considering *p* <0.2 (i.e. a less in 1 in 5 chance of the data having arisen randomly), as recommended for phase II screening trials by Rubinstein et al. (2005). In this way, if an experiment results in an outcome at the limit of interest there is a compelling argument for follow up experiments. Conversely, the effect size might be found to be substantial and the pilot study may have provided the p < 0.05 burden of proof needed and not at the cost of an excess of participants. Finally, this less stringent value for *α* will reduce the risk of using large numbers of animals in a first experiment to find that the effect size is near zero. For illustrative purposes the *n* needed to establish different effect sizes to an *α* rejection rate of *p* < 0.10 and *p* < 0.2 have been added to **Figure 1**.

**Figure 2:**
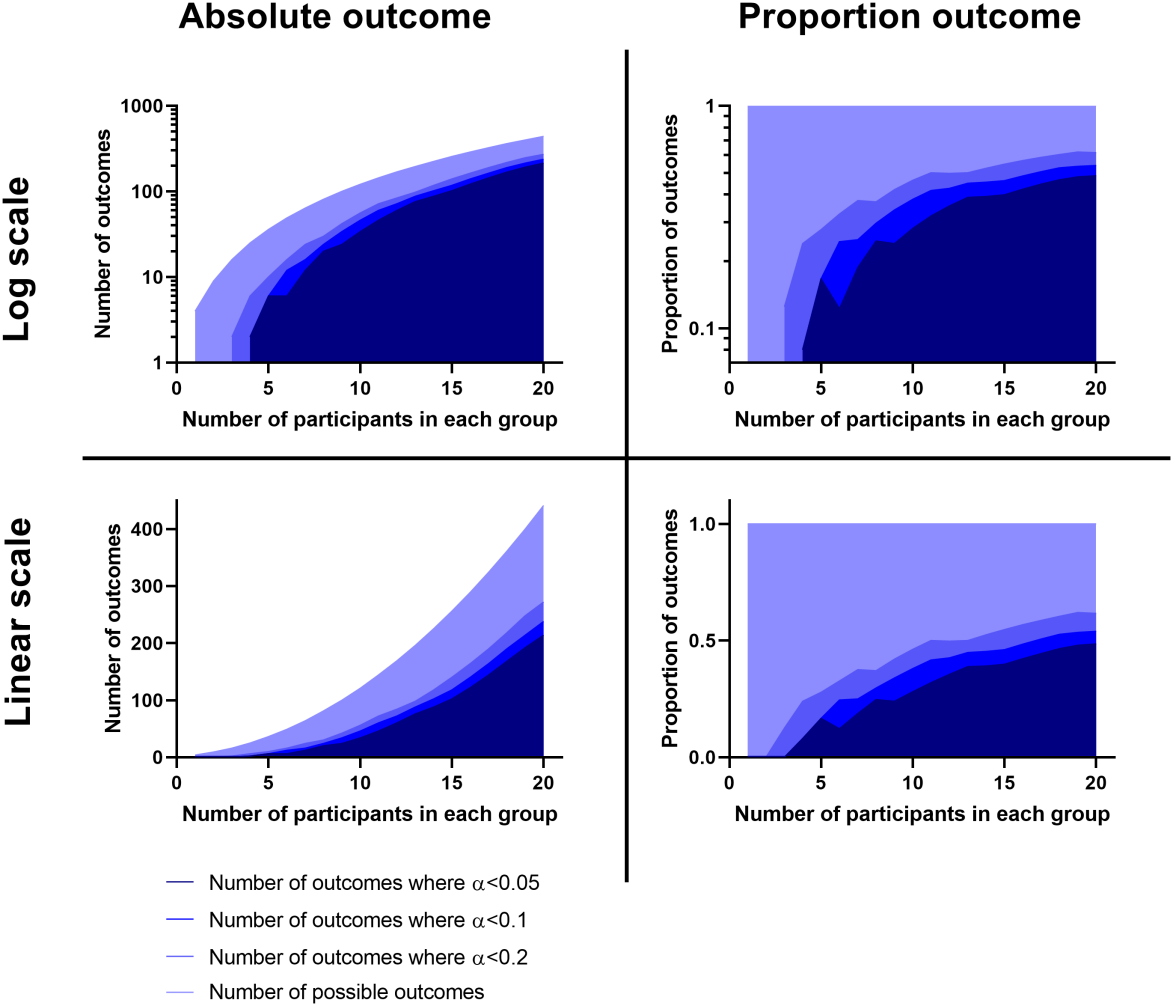
Plots showing the number and proportion of outcomes possible using a Fisher’s Exact test comparison in relation to how many of these outcomes would be beyond a statistically significant threshold. Calculations were made using the ‘fisher.test’ function from ‘stats’ base package in R V4.1.1 (R Core Team, 2021) and the graphs were prepared using the ‘xy plot’ function using single values for y in Graphpad PRISM V8.0 (San Diego). Left are plots of the actual number and right are these as a proportion. Above are these on a logarithmic scale, below are these on a linear scale.

In some experiments the infection will produce a binary outcome and time will be largely irrelevant. Here the data can be expressed as a contingency table where the number of treated and untreated survivors and non-survivors can be considered. Traditionally, these datasets are analysed using the Chi square test; however, this test assumes that all groups will have an expected value greater than 5. The Fisher’s exact test can handle frequencies as low as zero (Fisher, 1922). Power analysis tools exist for this statistical tool; however if the experimenter has limited prior understanding of the magnitudes of the outcome frequencies, then this tool will have little utility for pilot studies. Here we consider two things. Firstly, it is important to note that there are the same type of issues with survival proportion as there are with survival rates. In this way, there is benefit in focussing the experiment (where possible) towards the extreme in survival or non-survival. Second, the experimenter should consider only performing experiments where there is an acceptable probability of obtaining informative data. The plots in **Figure 2** show the *p*-values that are generated in different outcomes of a two group Fisher’s exact test. These plots can be treated in a similar manner to the survival proportions where the experimenter can choose a sample size which enables a “limit of interest” to be found where *p* < 0.2. This value for *α* is suggested for the same reasons as above, as a compromise of providing enough power to either: establish a very great difference, inform a future study or identify if there is little hope for an effect. It is clear looking at these plots that there are diminishing returns around *n* = 7 to 8 regarding increasing the numbers of participants. As such, it may be more difficult to justify an experiment where *n* is greater than 8 and the Fisher’s exact test will be the likely analysis technique unless the experimenter is interested in small effects. Moreover, this number also aligns with findings from the Allgoewer and Mayer simulation study which identified a minimum number of n=8 recommended for a categorical outcome for pilot animal experiments (Allgoewer and Mayer, 2017).

### Infectious burden and subject weight

**Figure 3:**
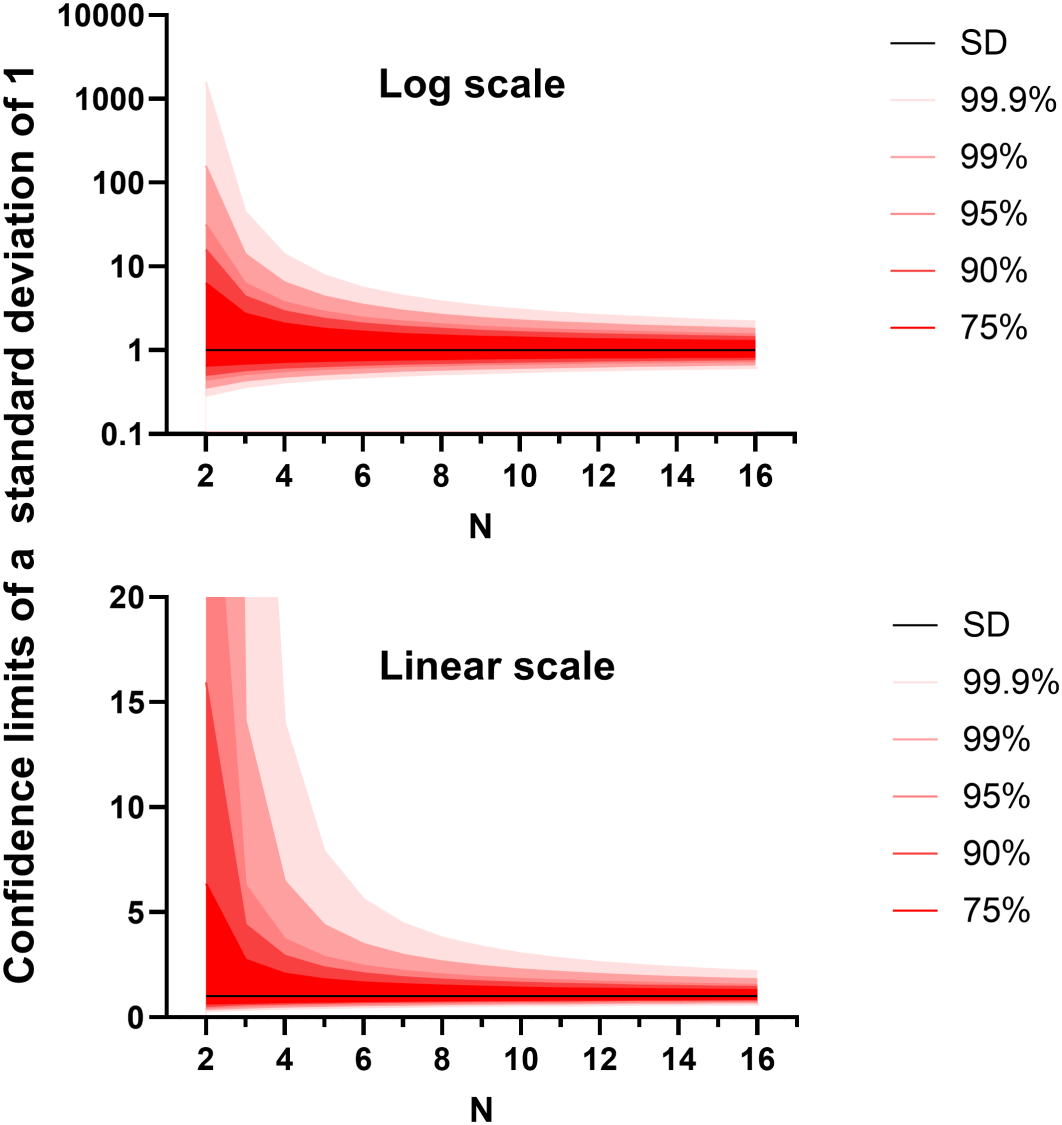
The confidence intervals for the standard deviation (set to a value of 1) generated from different sample sizes. The graphs were generated using Graphpad PRISM V8.0 (San Diego), using the ‘xy plot’ function using single values for y. The density function was used. Data were generated from first principles using Equation 3.5 on page 217 from the Handbook of Parametric and Nonparametric Statistical Procedures (Shedskin 2011). Above shows the relationship on a logarithmic scale and below shows the relationship on a linear scale.

In the other two experiment types that we often run, the primary metric for success is a continuous variable. These include animal weight measured through time and infectious burden within different organs. We have found little difference in the utility or outcome between using raw weights or weights as a proportion of initial conditions (data not shown). Bacterial (or viral) load is count data that can usually be converted into a normally distributed continuous variable through a simple logarithmic transformation. These data are often analysed by ANalysis Of VAriance (ANOVA). ANOVA considers how the data points vary from each other related to the explanatory variables (elements within the model to explain the data points). ANOVA tests can be used to determine whether there is a difference in means of the groups at each level of the independent variable. Fundamental to ANOVA is that, once the effect of the explanatory variable/s have been accounted for, the remaining variation approximately follows the normal distribution. This is mostly true for weight data and logarithm transformed microbial count data. It is this remaining variation that can be used to assess the probability that each explanatory variable is playing a role in the data. For this reason, an estimation of the “residual variation” is critical for future sample size calculation and a reliable estimate must be made by the pilot study. This estimate can inform the design of the future study and fulfil the obligation of a pilot study. It is important to account for as many experimenter-influenced factors as possible before taking the residual variation. Example analysis designs are discussed in the paragraphs below. However to stay on the question of making reliable estimates of residual variation, the standard deviation is an example of how population variability can be represented, where the data follows a normal distribution. The reliability of an estimate of the standard deviation can be found in the confidence intervals. As these confidence intervals are derived from a single positive figure, they can be calculated assuming a *χ*-distribution (Shedskin, 2011). We plotted different confidence intervals around a hypothetical standard deviation of one (**Figure 3**). Two things are made clear from this exercise. Firstly, a sample size of 2 has practically no value in predicting the true standard deviation. Secondly, the relation is exponential with diminishing returns for adding additional data points. We propose that for a pilot study, groups of between four and ten would be ideal depending on the number of organs/animal’s weights tested. Again, this number also aligns with the findings from the Allgoewer and Mayer simulation study of n=5-6 animals being reasonable for a continuous outcome (Allgoewer and Mayer, 2017).

The analysis plan then needs to consider what is being measured and in what ways can the variation be accounted for. This information can be used to build the ANOVA.

A simple example of an ANOVA would be microbial load measured at one time in independent animals treated with an intervention versus a control group. Here the sole explanatory variable is the *condition*, and its likely role in microbial load is estimated by the ANOVA by considering the variation generated by the treatment compared to the unaccounted for (residual) variation. The test provides a *p*-value indicative of the likelihood that the data might have arisen randomly. Where the likelihood is low (i.e. *p* < 0.05) the experimenter might reasonably consider that it was not random and the *condition* had an effect. These models can and should become more complicated when multiple organs are read. Here the statistical model can account for *subject* (i.e. if load is high in one organ it will likely be high in others) as well as *organ*. Once models become more complicated, they can also consider how explanatory variables interact together. In an example where data has been gathered from different *organs*, in animals treated in different *conditions*, the interaction term *organs;condition* can be considered. To paraphrase this interaction considers the question of whether the *conditions* alter the microbial load equally in the different *organs*.

A further example would be a typical weight change infection study. Here the model will consider the explanatory variables of: *time* (typically weight decreases as the infection takes hold but can then recover), *subject* (some variation can be accounted for by considering that each data point is related to its previous and future data points) and *condition* (the experimentally altered factor). For the reason that the *condition* is only likely to affect weight when the infection also has an effect, the interaction of *time* and *condition* (*time:condition*) is the key readout of the analysis. This interaction represents the effect of the experimentally altered factor on “weight change” not just “weight”.

One general consideration regarding pilot studies is the analysis plan. In the performance of powered studies, a clear understanding of the desired comparisons are critical for preventing issues related to multiple testing (or “cherry picking”). This is still critical for pilot studies despite the fact that the main desired outcome is an estimate of variability and effect size for powering future studies. This primary purpose does not insulate the pilot study from the potential errors associated with multiple testing. Cherry picking is the process where the investigators perform all possible comparisons and choose to report the probabilities that confirm the hypothesis without accounting for the enhanced likelihood for false positives associated with the total number of tests performed. The experimenter should consider and pre-specify the analysis plan. If multiple testing is required, then adjustments should be made accordingly, e.g. by way of a Bonferroni correction which is easy and simple to implement. Further guidance and discussion on multiple testing, including alternative multiple testing approaches, are available (Holm, 1979; Hommel, 1988; Hochberg, 1988; Wright, 1992; Benjamini and Hochberg, 1995; Shaffer, 1995; Sarkar and Chang, 1997; Sarkar, 1998; Benjamini and Yekutieli, 2001).

**Figure 4:**
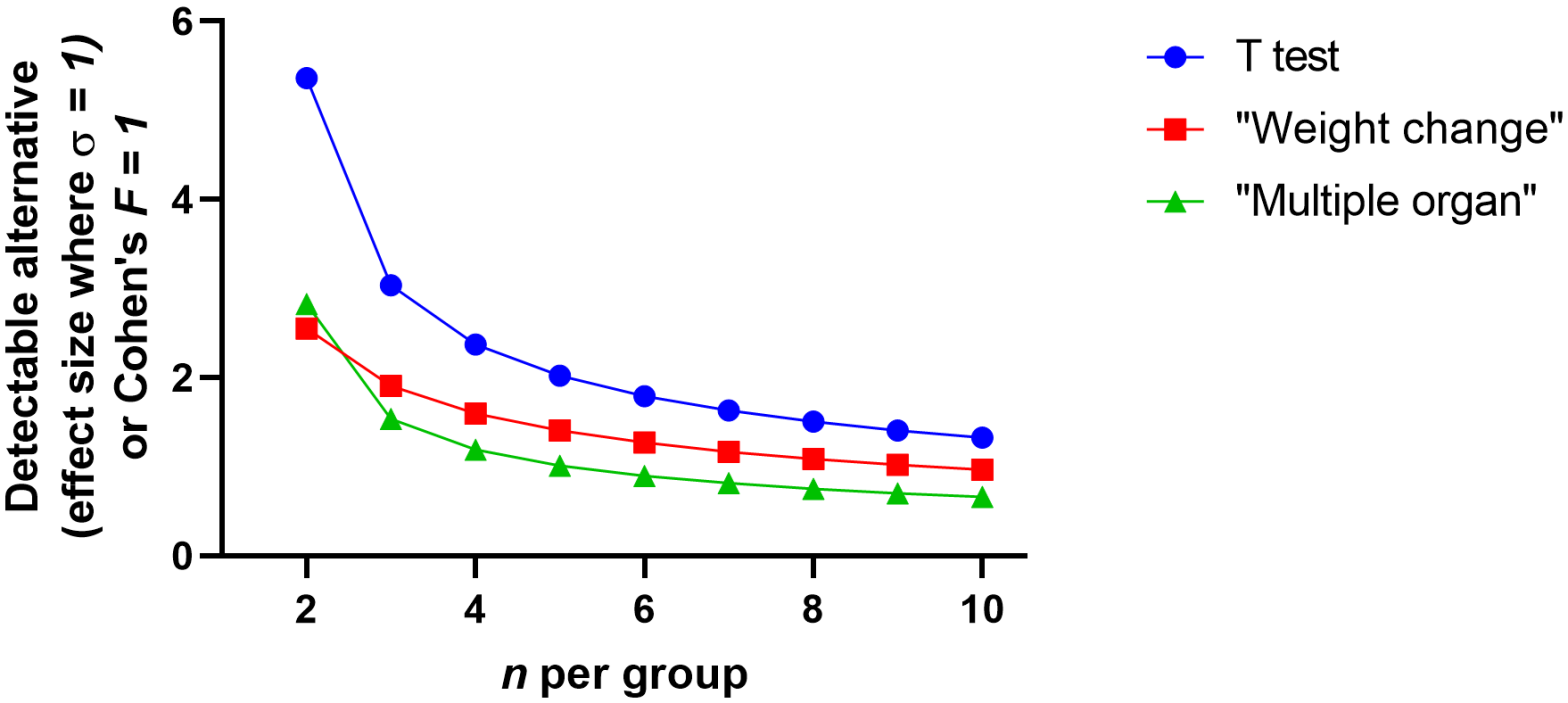
The detectable difference between two independent groups where n is small (2 to 10) where an assumed standard deviation of 1 or for analysis of variance Cohen’s F is used. The graphs were generated using Graphpad PRISM V8.0 (San Diego), using the ‘xy plot’ function using single values for y. Data were manually transferred from power software: the T-test power analysis was performed using PS: Power and Sample Size Calculation V3.0 (https://biostat.app.vumc.org/wiki/Main/PowerSampleSize). The software uses the method described by Dupont et al. (Dupont and Plummer, 1990) and power was set to 80% and alpha set to 5%. The analysis of variance power analysis was performed using G*Power V3.1.9.7 (https://www.psychologie.hhu.de/arbeitsgruppen/allgemeine-psychologie-und-arbeitspsychologie/gpower). The software uses the method described by Cohen (1988) with power was set to 80%, alpha set to 5% and sphericity correction set to 1. The weight change experiment supposes 14 time points and the interaction being the point of interest. The multiple organ experiment assumes 3 organs and the between factor component being the point of interest.

The statistical advantage of using a more complicated analysis design and multiple measurements is shown in **Figure 4**. This figure suggests that the ratio of detectable effect and variation against *n* shows the small values for *n* used in pilot studies would be only likely to find the largest of effects statistically probable. First, when we consider the detectable effect size of the *t*-test, we see that the pilot studies will only be able to identify very substantial effect sizes related to variation. However, this can be negated by blocking and multiple measures (i.e. the inclusion of further explanatory variables discussed above). For example, in the weight change experiment, each experimental subject has multiple measurements. Animal weight should be consistent at the start of the experiment and diverge as the interventions take effect. The analyst can use several options. The single comparison (*t*-test) route has poor prospects; either the analyst chooses a single time point to measure where they believe the weight difference might be at its greatest or all time points are taken and multiple tests are performed accounting for increased false positive rate of multiple tests. Both single comparison options are weak. ANOVA has the advantage of considering the whole data set whilst also enabling to account for other explanatory variables. Due to weight divergence being the key metric for difference, the interaction value in a repeated measure ANOVA becomes the test statistic of interest. For illustrative purposes consider a 14 day experiment with a sphericity correction of 1 (**Figure 4**). For ANOVA, the Cohen’s *F* value is the nearest equivalent to the t-test effect size. Broadly the Cohen’s *F* is a ratio of the desired effect size against the other variability in the system. It is clear here that the collective analysis has greater statistical power and this might be obvious when we consider that more data is used. When considering the infectious load experiment a similar conclusion is derived. The single comparison route either necessitates the capture of a single foci of infection or multiple points and false positive down adjustments of significance. ANOVA can account for foci of infection (such as organ) or multiple data points through the natural history of disease. Again for illustrative purposes we consider a 3 organ experiment and find the minimum discoverable effect size is better than that of the *t*-test, even before multiple testing adjustments would need to be performed. It is clear then that, for pilot studies, the analyst should use analysis techniques that collectivise gathered data. Moreover, this might increase statistical power sufficiently that the pilot study may be enough to provide sufficient proof against the hypothesis and be able to adjust for potential confounding factors.

## Conclusion

**Figure 5:**
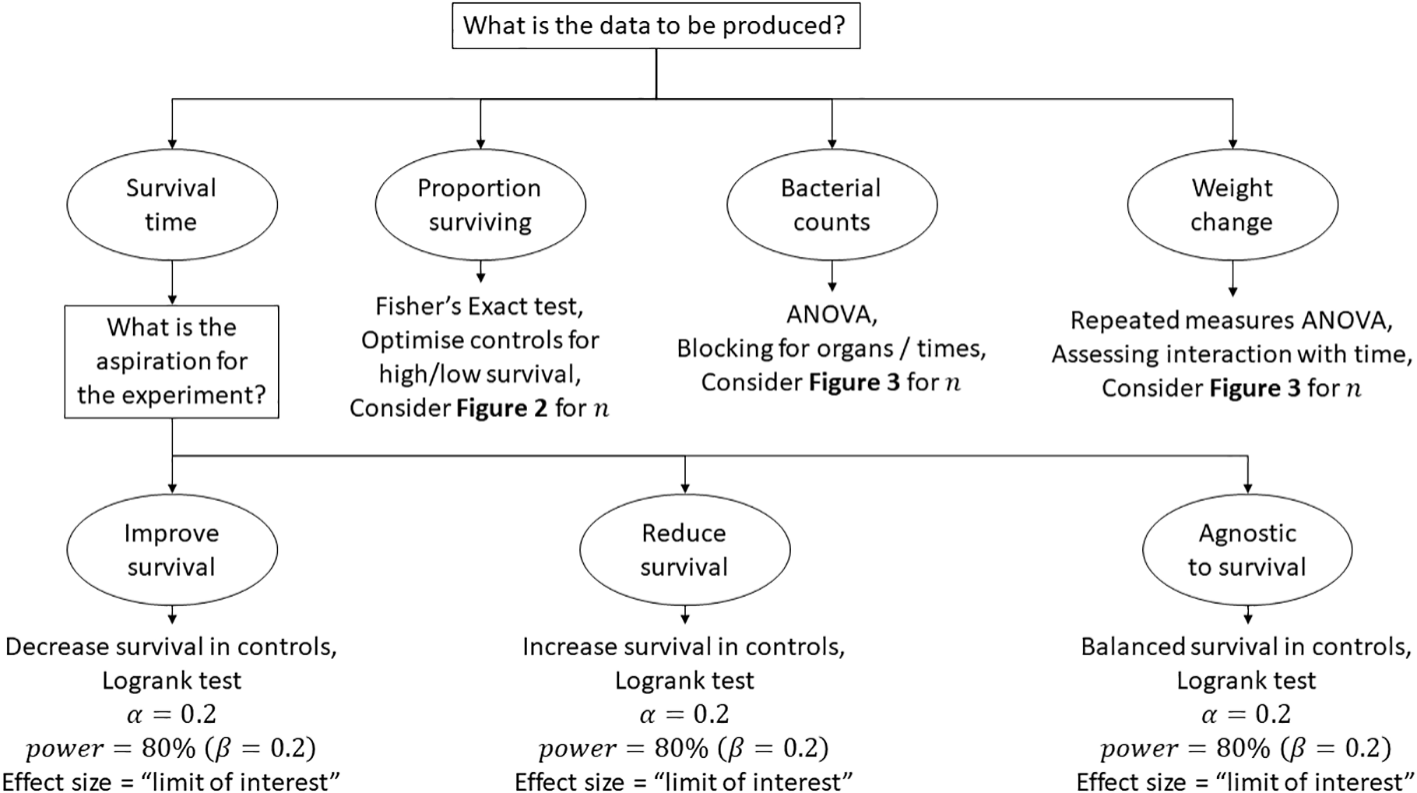
A flow diagram to aid in considering the n for a pilot infection study.

**Figure 5** is a map of the thought process behind the design of a pilot study when a hypothesis is considered and there is little to no evidence of the expected outcome. We hope that these thoughts might prove beneficial and would actively encourage debate regarding this largely neglected subject. To reiterate, it would be advantageous ethically to attain a better understanding on the requirements for animal experiments that lie in the “grey area” of first attempt of concept often referred to as “pilot studies”. It is likely that a significant proportion of animal academic research lies in this grey area. These considerations that we have outlined specifically consider infection research; however, they may also be directly applicable to other avenues of research.

## Data availability statement

Only example data has been produced using freely available software. This can be provided on request or the interested party can easily reproduce these datasets.

## Author contributions

This manuscript was written by both authors. Figures and analysis were prepared by Thomas Laws. All authors contributed to the article and approved the submitted version.

## Funding

The author’s time was funded by the UK, Ministry of Defence.

## Acknowledgments

We thank the invaluable discussion with our colleagues Daniel Silk and Michelle Nelson. We would also like to thank the reviewers for their helpful comments, which have enabled us to improve the paper accordingly.

## Conflict of interest

The authors declare that the research was conducted in the absence of any commercial or financial relationships that could be construed as a potential conflict of interest.

## Publisher’s note

All claims expressed in this article are solely those of the authors and do not necessarily represent those of their affiliated organizations, or those of the publisher, the editors and the reviewers. Any product that may be evaluated in this article, or claim that may be made by its manufacturer, is not guaranteed or endorsed by the publisher.
